# TNFR1 and TNFR2, Which Link NF-κB Activation, Drive Lung Cancer Progression, Cell Dedifferentiation, and Metastasis

**DOI:** 10.3390/cancers15174299

**Published:** 2023-08-28

**Authors:** Gongping Shi, Yinling Hu

**Affiliations:** Cancer Innovation Laboratory, Center for Cancer Research, National Cancer Institute, National Institutes of Health, Frederick, MD 21702, USA; gongping.shi@nih.gov

**Keywords:** TNFR1, TNFR2, NF-κB, lung squamous cell carcinoma, lung adenocarcinoma

## Abstract

**Simple Summary:**

This review discusses new findings for the roles of TNFR1 and TNFR2 in the development of aggressive lung squamous cell carcinoma and lung adenocarcinoma by diverse signaling pathways in lung epithelial tumor cells and leukocytes participating in immunosuppressive tumor microenvironment generation in animal models. These defined events were consistently identified in human lung SCCs and ADCs. The components in these molecular mechanisms may be considered potential therapeutics for lung cancer.

**Abstract:**

TNFR1 and TNFR2, encoded by *TNFRSF1A* and *TNFRSF1B*, respectively, are the most well-characterized members among the TNFR superfamily. TNFR1 is expressed in most cell types, while TNFR2 has been reported to be preferentially expressed in leukocytes. Lung cancer remains the leading cause of cancer mortality worldwide but TNFRs’ activities in lung cancer development have not been fully evaluated. Recently, overexpressed TNFR1 was reported in a large proportion of human lung squamous cell carcinomas. Increased TNFR1 coupled with increased UBCH10 caused lung SCC cell dedifferentiation with epithelial–mesenchymal transition features and the metastasis in a combined spontaneous lung SCC and TNFR1 transgenic mouse model. UBCH10, an E2 ubiquitin-conjugating enzyme that is an oncogene, increased Sox2, c-Myc, Twist1, and Bcl2 levels. Increased TNFR1 upregulated UBCH10 expression by activating c-Rel and p65 NF-κB. Lung SCC patients overexpressing *TNFRSF1A* and one of these target genes died early compared to lung SCC patients expressing lower levels of these genes. Recently, we also revealed that TNFR2 was required for lung adenocarcinoma progression, delivering a signaling pathway of TNF/TNFR2/NF-κB-c-Rel, in which macrophage-produced ROS and TNF converted CD4 T cells to Foxp3 Treg cells, generating an immunosuppressive tumor microenvironment and promoting lung ADC progression. In human lung ADC cohorts, *TNFRSF1B* expression was highly correlated with *TNF*, *FOXP3*, and *CD4* expression. Of note, TNF stimulated the activities of TNFR1 and TNFR2, two membrane-binding receptors, which accelerate tumorigenesis through diverse mechanisms. This review focuses on these new findings regarding the roles of TNFR1 and TNFR2 in lung SCC and ADC development in humans and mice, and highlights the potential therapeutic targets of human lung cancers.

## 1. Introduction

Human lung cancer is still a leading cause of cancer mortality. Non-small cell lung carcinoma (NSCLC), which represents 80–90% of human lung cancer, includes lung adenocarcinoma (ADC) (50%), squamous cell carcinoma (SCC) (35%), and large cell carcinoma (15%) [1]. Lung SCC and ADC derive from different lung epithelial cells and exhibit distinct causes, morphology, and gene expression patterns [1,2,3,4]. Approximately 30–40% of human lung ADCs contain activated *KRAS* mutations at its residue 12, 13, or 61 [3,4]. Activated *Kras* mutations or chemical urethane carcinogen mediate lung ADC and many upregulated oncogenes and loss of tumor suppressors promote *Kras*- or chemical carcinogen-initiated lung ADCs [2,5,6]. On the other hand, activated *KRAS, HRAS*, and *NRAS* mutations were found in very few human lung SCC cases (cBioPortal for Cancer Genomics, lung cancer cohorts). To date, three distinct mechanisms for initiating lung SCC development in mice have been reported: (1) combined *p53* deletion with *Lkb1* or *Kmt2d* deletion [7,8]; (2) combined deletion of *Pten* and *Cdkn2ab* with overexpressed Sox2 [9]; and (3) kinase-dead *Ikka* knock-in (*KA/KA*) associated with pulmonary inflammation and IKKα reduction [1]. All these lung SCC animal models require the loss of one or two tumor suppressors. Recently, we reported that overexpressed tumor necrosis factor receptor 1 (TNFR1) induced lung SCC cell dedifferentiation and metastasis to the liver in *KA/KA* mice [10] and that TNFR2 in CD4 T cells was required for generating an immunosuppressive tumor microenvironment (iTME) for promoting lung ADC progression in *Kras^G12D^;Ikka^∆LU^* mice [11].

**Figure 1:**
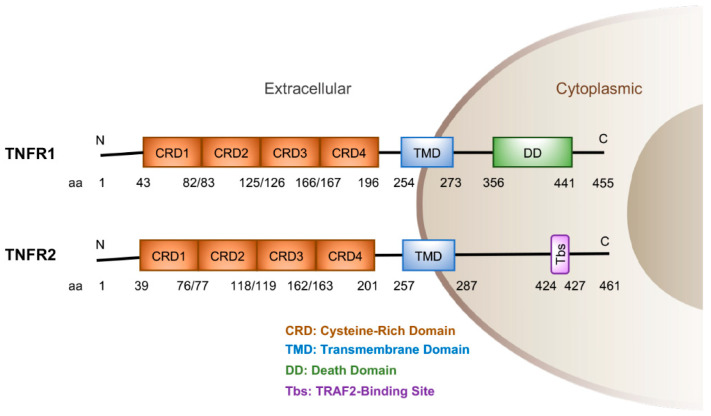
Human TNFR1 and TNFR2 protein structures and amino acid (aa) numbers. N, N-terminal region; C, C-terminal region.

Members of the TNFR superfamily contain multiple cysteine rich domains in the N-terminal region and a transmembrane domain in the middle region (Figure 1). TNFR1, a 455-amino-acid-polypeptide, contains a death domain (DD) in its C-terminal region. TNFR2, containing 461 amino acids, does not contain this death domain, but, instead, includes a TRAF2 binding site (Tbs) at the C-terminal region. These indicate that TNFR1 and TNFR2 lead to different pathways for distinct events and activities based on specific cell types and their internal domains. TNF cytokine can stimulate TNFR1 and TNFR2 activities [10,11]. For example, TNF-activated TNFR1 induces IKK, which is composed of IKKα, IKKβ, and IKKγ, and NF-κB activation [12,13], although the individual physiological activity of these molecules in this pathway shows linear functions or diverse effects, depending on the cell types [14]. In this review, we mainly discuss the newly identified physiological roles and mechanisms of TNFR1 and TNFR2 in lung SCC and lung ADC development in humans and mice. These new findings highlight the possible therapeutic targets for lung cancer.

## 2. TNFR1 Overexpression Promotes Lung SCC Cell Dedifferentiation and Metastasis

### 2.1. Background

The skin, lungs, esophagus, oral cavity, and uterine tubes are protected by a stratified or pseudostratified epithelial structure that is composed of several cell layers from the undifferentiated basal cells to functionally and terminally differentiated cells that protect the organs [1,15,16]. SCC derives from the basal epithelial cells or the epithelial cells with stemness properties, which can dedifferentiate or proliferate in these organs with loss of tumor suppressors or overexpression of oncogenes. TNFR1 is ubiquitously expressed in many types of cells in the body. The roles of TNF and TNFR1 have been examined for chemical carcinogen-induced skin carcinogenesis [17,18]. The *7,12-dimethylbenz[a]anthracene* (DMBA) treatment induces *Hras* mutations at amino acid residue 12, 13, or 61, and 12-*O*-tetradecanoylphorbol-13-acetate (TPA) elicits inflammation in the skin. DMBA/TPA-induced skin tumorigenesis was dampened in *Tnfr1^-/-^* or *Tnf^-/-^* mice compared to WT mice [17,18]. Double *Tnfr1^-/-^;Tnfr2^-/-^* mutant mice inhibited DMBA/TPA-induced skin carcinogenesis in a bacterial count-dependent manner, which required a TLR5 pathway [19]. Furthermore, TNFR1 was required for obesity-induced tumor promotion in a diethylnitrosamine (chemical carcinogen)-initiated liver carcinogenesis setting [20]. Thus, TNFRs promote carcinogen-induced skin and liver tumor development. However, the role of TNFR1 in lung cancer has not been intensively investigated yet.

### 2.2. TNFR1 in Human Lung SCC

Lung SCC frequently occurs in the upper and center of the lungs and derives from the basal epithelial cells that are able to proliferate or dedifferentiate. NSCLC cohorts from The Cancer Genome Atlas Program (TCGA) with increased *TNF* expression showed significantly reduced survival compared to NSCLC patients with reduced *TNF* [21]. Increased expression of *TNFRSF1A* was associated with a reduced trend in lung SCC patients’ survival (media, Logrank *p*-value = 0.08, OncoLnc). In addition, immunohistochemical (IHC) staining showed increased TNFR1 expression in a large proportion of human lung SCCs [10]. Consistently, analyses with reverse-transcription polymerase chain reaction showed increased TNFR1 mRNA in a large proportion of human lung SCCs. These data suggest that increased TNF and TNFR1 levels may facilitate lung SCC development. Thus, there is an urgent need to identify TNFR1′s targets in human lung SCCs. We then hypothesize that patients with lung SCCs expressing a combination of increased *TNFRSF1A* and one of its targets may show significantly reduced survival. This remains to be confirmed by animal models.

### 2.3. A Lung SCC Mouse Model

Tumor suppressor genes of *TP53*, *RB1*, and *LKB1* are frequently mutated in human lung SCCs [3]. A single deletion of either of these tumor suppressor genes does not induce spontaneous lung SCC in mice, but mice lacking double *Lkb1* and *p53* develop spontaneous lung SCCs or mixed SCCs and ADCs [7]. Of note, we previously reported that *KA/KA* mice, which express a mutated IKKα protein containing a single mutation at amino acid 44, an ATP binding site, with a replacement of an alanine (A) residue from a lysine (K), developed spontaneous lung SCCs. This KA/KA mutation destabilized IKKα proteins in the lung epithelial cells with increased ages and was associated with increased macrophage infiltration in mice, which contributed to spontaneous lung SCC development in *KA/KA* mice [1]. Reintroducing transgenic IKKα, in which an IKKα cDNA is controlled by a keratin 5 (K5) promoter, or depleting macrophages, diminished lung SCC development [1]. The lung SCCs derived from *KA/KA* mice exhibited the classical SCC features with keratin pearls and increased K5, p63, and Ki67 positive cells, and expressed reduced p53, Rb, and LKB1 tumor suppressors but elevated EGFR activity, ERK activity, c-Myc, Trim29, Rhov, Nanog, and ROS1 levels [1]. In addition, the pulmonary infiltrating macrophages within *KA/KA* lung SCCs expressed increased inducible nitric oxide synthase (iNOS), which is encoded by *Nos2*, and many cytokines including IL-3, IL-21, IL-22, and IL-24 [22,23]. *KA/KA;Nos2^-/-^* mice inhibited lung SCC development. Transfer with *KA/KA* and *KA/KA;Nos2^-/-^* bone marrow (BM) to irradiated wild-type (WT), *KA/KA*, or *KA/KA;Nos2^-/-^* mice demonstrated that iNOS in tumor cells and macrophages contributes to lung SCC development. iNOS levels are very low in normal cells. TNF is a strong inducer of iNOS expression [22], which provides a connection between iNOS and TNF/TNFRs in tumorigenesis.

### 2.4. TNFR1 Induction in Lung SCC Development in Human and Mice

**Figure 2:**
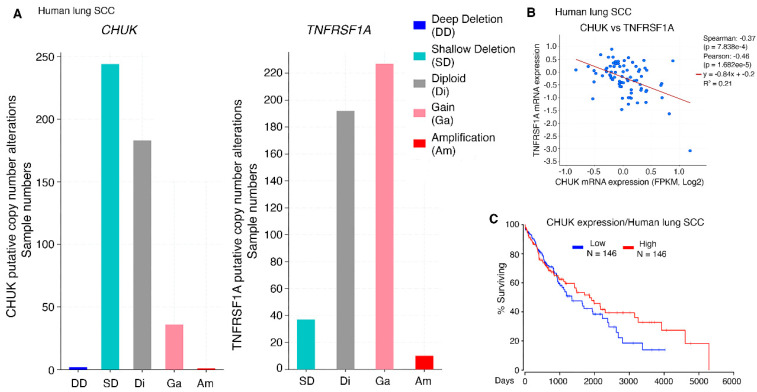
*TNFRSF1A* and *CHUK* correlations in human lung SCCs (cBioPortal, TCGA, PanCancer Atlas). (**A**). Distributions of *CHUK* (left) and *TNFRSF1A* (right) expression alterations in human lung SCC numbers. (**B**). Correlation of *TNFRSF1A* and *CHUK* expression in human lung SCCs (cBioPortal, CPTAC, Cell 2021). (**C**). Survival rate of human lung SCC patients with the top 30% and the bottom 30% of *CHUK* expression levels (OncoLnc), Logrank *p*-value = 0.3.

Increased TNFR1 protein and mRNA levels were detected in *KA/KA* lung SCCs that express reduced IKKα [10]. Although the mutation rate of *CHUK* in human lung SCC was low [3], its expression levels were reduced in a large proportion of human lung SCCs, which were reciprocal to *TNFRSF1A* expression patterns in human lung SCCs (Figure 2A). Indeed, the expression levels of *CHUK* and *TNFRSF1A* were totally opposite in human lung SCCs (Figure 2B). Again, high IKKα levels were correlated with an increased survival for lung SCC patients (Figure 2C). Downregulation of IKKα in human cancers is associated with the DNA methylation of the *CHUK* gene [24]. In addition, treatment with TNF cytokine downregulates IKKα levels in human SCC cell lines [16]. These suggest that multiple pathways determine IKKα expression and stability. Together, this *KA/KA* animal lung SCC model represents a relevant human lung cancer setting and the findings from these studies will provide therapeutic value.

### 2.5. Increased TNFR1 in Lung SCC Cells Drives Cancer Stemness, Dedifferentiation, and Metastasis

IHC detected significantly increased TNFR1 in human lung SCC cells, suggesting an intrinsic activity of TNFR1 in lung SCC cells. To determine an overexpressed TNFR1′s role, we generated a transgenic mouse strain overexpressing Tg-K5.TNFR1, in which TNFR1 cDNA is controlled by a K5 promoter [10,25]. The lungs of Tg-K5.TNFR1 young mice are comparable to those of WT mice, but the lungs of the transgenic mice at 10 months of age show minor increased epithelial proliferation with slightly inflammatory cell infiltration. Furthermore, lung SCCs were detected in three-month-old *K5.TNFR1;KA/KA* mice, while 5–10-month-old *KA/KA* mice start to show lung SCCs, suggesting that overexpressed TNFR1 promoted lung SCC initiation. In addition, *K5.TNFR1;KA/KA* lung SCCs display dedifferentiated features and metastasize to the liver [10], suggesting that TNFR1 promotes lung SCC initiation, dedifferentiation, and metastasis.

To determine the mechanism by which intrinsic TNFR1 modulates tumor cell fates, we used Scal1 and CD24 stem cell markers to isolate KAL^LU+^ lung SCC cells with high stemness (Scal1^high^CD24^low^) features and KAL^LU-^ cells with low stemness (Scal1^low^CD24^high^) features from a previously reported parental KAL^LU^ lung SCC cell line derived from *KA/KA* lung SCCs [1,10]. Importantly, Scal1^high^CD24^low^KAL^LU+^ cells highly expressed TNFR1 and Sox2 compared to Scal1^low^CD24^high^KAL^LU-^ cells [10]. Silencing TNFR1 downregulated Sox2 levels in KAL^LU+^ cells. Sox2, a stem cell marker, is highly expressed in human lung SCCs [26]. Thus, TNFR1 levels are associated with cancer stemness potential. Consistently, the lung tumor burdens were significantly higher when induced by the KAL^LU+^ cells via tail vein injection than by the KAL^LU-^ cells in WT and *KA/KA* mice, and KAL^LU+^ cancers, but not KAL^LU-^ cancers, metastasized to the liver [10]. KAL^LU+^ cell-derived lung spindle cell carcinomas, a final stage of SCCs, showed dedifferentiated and epithelial–mesenchymal transition (EMT) features with E-cadherin loss and K5 expression reduction [10,27]. KAL^LU-^ cell–derived well-differentiated lung SCCs expressed high levels of K5 and low levels of involucrin, an intermediate differentiation marker. Silencing TNFR1 in KAL^LU+^ cells reduced the cell-derived tumor burdens and converted the spindle cell carcinomas to well-differentiated SCCs [10]. These results suggest that a high TNFR1 level drives dedifferentiation and metastasis of SCC cells. Interestingly, human and mouse lung SCCs contain full-length and cleaved TNFR1 proteins [10]. Cell-surface membrane-binding and Golgi-associated TNFR1s exist in cells [28,29]. The functions of these different forms of TNFR1 in tumorigenesis remain to be revealed.

While working to elucidate the molecular bases for the different oncogenic activities in KAL^LU+^ and KAL^LU-^ cells, we identified higher levels of Sox2, c-Myc, Twist1, Bcl2, and UBCH10 in KAL^LU+^ cells than in KAL^LU-^ cells. Silencing TNFR1 in KAL^LU+^ cells downregulated the levels of these proteins. UBCH10, an E2 ubiquitin-conjugating enzyme, which is encoded by *UBE2C*, is an oncogene. Its targets include Sox2, c-Myc, Twist1, and Bcl2. Overexpressed UBCH10 causes chromosome missegregation and results in multiple types of spontaneous tumors [30,31,32,33]. Of note, TNF treatment of KAL^LU+^ cells elevated UBCH10 and downregulated E-cadherin and K5 levels, whereas, knocking down UBCH10 elevated K5 and E-cadherin levels, indicating that TNF/TNFR1 and UBCH10 lead a program that switches differentiation and dedifferentiation or EMT of SCC cells, which determines a tumor’s regression or progression. Indeed, silencing TNF impaired KAL^LU+^ cell–derived lung tumor development. Importantly, patients with lung SCCs expressing increased TNFRSF1A with TWIST1 or UBE2C died much earlier than lung SCC patients expressing low levels of these genes [10]. Thus, these molecular alterations are relevant to human cancer development.

Consistently, we used the same methods to isolate cancer stem cells from human H520 SCC cells with CD24 and CD44 markers [10]. TNFR1 expression levels were higher in CD24^low^CD44^+^H520 cells than in CD24^high^CD44^+^H520 cells [10]. Subcutaneously injected CD24^low^CD44^+^H520 cells developed tumors at the injection location and metastasis to the lungs but CD24^high^CD44^+^H520 cells did not develop tumors in these mice. These results indicate that TNFR1 shows similar activities in human and mouse lung SCCs.

### 2.6. TNFR1 Levels Correlate with Induced NF-κB Activity That is Required for UBE2C/Ube2c Promoter Activity

Previously, we reported that the CD45^-^CD31^-^ lung epithelial cells isolated from *KA/KA* mice showed increased NF-κB activity compared to lung epithelial cells isolated from WT mice and that elevated NF-κB activity was correlated with decreased IKKα levels due to this *KA/KA* mutation that destabilizes IKKα proteins in *KA/KA* lung epithelial cells [1]. In addition, TNF treatment induced higher levels of nuclear p65 and p50 in parental KAL^LU^ cells than in M2C cells that are mouse transformed lung epithelial cells. Furthermore, the induced nuclear c-Rel, p65, and p50 NF-κB levels were higher in KAL^LU+^ stem cells expressing high TNFR1 levels than in KAL^LU-^ cells expressing low TNFR1 levels. Research has noted that TNF promotes a cancer stem-like cell property [34]. Thus, TNFR1 levels correlate with increased NF-κB activities in response to cytokine stimulation and elevate cancer stem-like cell properties.

The c-Rel DNA-binding motifs are found in the promoter region of *Ube2c*/*UBE2C* [10,35]. The p65 and c-Rel enrichments were found with the *Ube2c*/*UBE2C* gene in mouse KAL^LU+^ and human SW900 SCC cells, which were associated with increased H3K27ac, an activated transcriptional marker [10]. TNF treatment, indeed, increased the levels of NF-κB enrichment on the *Ubec2c* promoter. Interestingly, the IKKα enrichment also formed the complex with the *Ube2c* promoter, but TNF treatment reduced levels of the IKKα enrichment. The enrichment levels of NF-κB and IKKα were reciprocal. Consistently, IKKα repressed UBCH10 protein and mRNA levels in KAL^LU+^ cells, although IKKα did not significantly repress TNFR1 levels. Overexpressed IKKα repressed KAL^LU+^ cell–derived tumor burdens in mice [10]. Clearly, in this case, increased NF-κB activity was correlated with high TNFR1 levels in KAL^LU+^ cells compared to KAL^LU-^ cells after TNF treatment, whereas IKKα inhibited tumorigenesis through repressing downstream targets of overexpressed TNFR1 (Figure 4A). Overall, TNF/TNFR1/NF-κB regulated UBCH10, which further mediated TWIST1, Sox2, and Bcl2 levels, contributing to SCC dedifferentiation, EMT, and metastasis (Figure 4A). In addition, patients with lung SCCs expressing high levels of *TNFRSF1A* and *REL* died earlier than patients with lung SCC expressing low levels of these genes [10]. Intriguingly, a report showed that transgenic Tg-K5.mIκBα mice, in which a mutated IκBα at its serine residues 32 and 36 is controlled by the K5 promoter, developed severe skin hyperplasia and inflammation [36]. The skin phenotype was rescued by *Tnfr1* deletion in Tg-K5.mIκBα;*Tnfr1^-/-^* mice [37]. Furthermore, our unpublished data suggest that this mIκBα, an un-degraded form, may interact with IKKα, impairing normal IKKα activity in the skin cells [14,15,38,39], which may contribute to the skin phenotypes in K5.mIκBα mice.

## 3. Role of TNFR2 in Lung Adenocarcinoma Progression

### 3.1. Background

Lung ADC, a major type of human lung cancer, derives from lung type II alveolar epithelial cells [2]. TCGA human cancer database analysis revealed that increased *TNFRSF1A* is correlated with significantly reduced survival of lung ADC patients [21]. TNFR1 is an upstream regulator of NF-κB. Increased NF-κB activity promotes *Kras^G12D^*-initiated lung ADC development with accelerating *p53* loss [40]. In contrast, treatment with a NF-κB inhibitors (bortezomib and bay-117082) repressed lung ADC development [41]. TNFR1, as an upstream transducer of NF-κB, may use the NF-κB pathway to promote lung ADC development. The internal domains of TNFR1 and TNFR2 are different at their C-terminal regions (Figure 1). Through the DD, TNFR1 stimulation at the plasma membrane forms complex I or complex II, which leads to diverse consequences. Complex I, containing TNFR1, TRADD, RIP1, TRAF2, and cIAP1, activates NF-κB and promotes cell survival. Complex II further recruits TRADD and RIP1 associated with FADD and caspase-8, resulting in cell death [42,43]. These suggest that TNF/TNFR1/NF-κB is sufficient to promote lung ADC as well as SCC development. Consistently, our unpublished data detected increased cIAP1 levels in some human lung SCCs. Because complex I and complex II lead to opposite results, it will be important to test whether alterations in components of TNFR1 complex I and TNFR II may change lung cancer fates in the future [42,43]. If so, the findings will provide new insight into lung cancer therapy. Indeed, patients with lung ADCs overexpressing *TNFRSF1A* died earlier than lung ADC patients expressing low *TNFRSF1A* levels [21].

TNFR2 shows the cell-type specific activities, such as those leukocytes, compared to TNFR1. *Tnfr2^-/-^* mice have a defect in Foxp3^+^ Treg cell development [44]. Foxp3, a transcriptional factor, is a crucial regulator for Treg cell induction [45]. There are three highly conserved noncoding sequences (CNS), CNS1 to CNS3, in the *Foxp3* locus, which are required for Foxp3 expression [45]. NF-κB c-Rel binds to CNS3 to induce Foxp3 expression [45]. Mice with a deletion of *Nfkb p65* or *c-Rel* in Foxp3^+^ cells show a defect in Treg cell induction, resulting in autoimmune diseases [46,47]. Increased NF-κB-induced Treg cell numbers generate an iTME for tumor promotion. Foxp3^+^ Treg cells suppressed antitumor T-cell responses, facilitating *Kras^G12D^*-mediated lung ADC development [48]. Thus, we examined whether TNFR2 in CD4 T cells via NF-κB is required for Treg induction to generate the iTME for lung ADC progression [11].

### 3.2. TNFR2 in CD4 T Cells Is Required for Generating an iTME with Increased Treg Numbers

Approximately 35–40% of human lung ADCs contain activated mutations at *KRAS* residue 12, 13, or 61 [4] (human lung ADC cohorts, cBioPortal for Cancer Genomics). *Kras^G12D^* is a lung ADC driver in mice [6]. *Ikka* deletion or reduction in lung cells with activated *Kras^G12D^* promoted *Kras^G12D^*-mediated ADC progression in *Ikka^∆LU^;Kras^G12D^* and *Ikka^∆LU/+^;Kras^G12D^* mice compared to *Kras^G12D^* mice [2]. Consistently, *Ikka* deletion in type II alveolar epithelial cells in the lungs, which was mediated by a pulmonary-associated surfactant protein C Cre, promoted urethane-induced lung ADC [5]. In the two models, although *Ikka* was ablated, NF-κB activity was not impaired in lung ADC epithelial cells.

**Figure 3:**
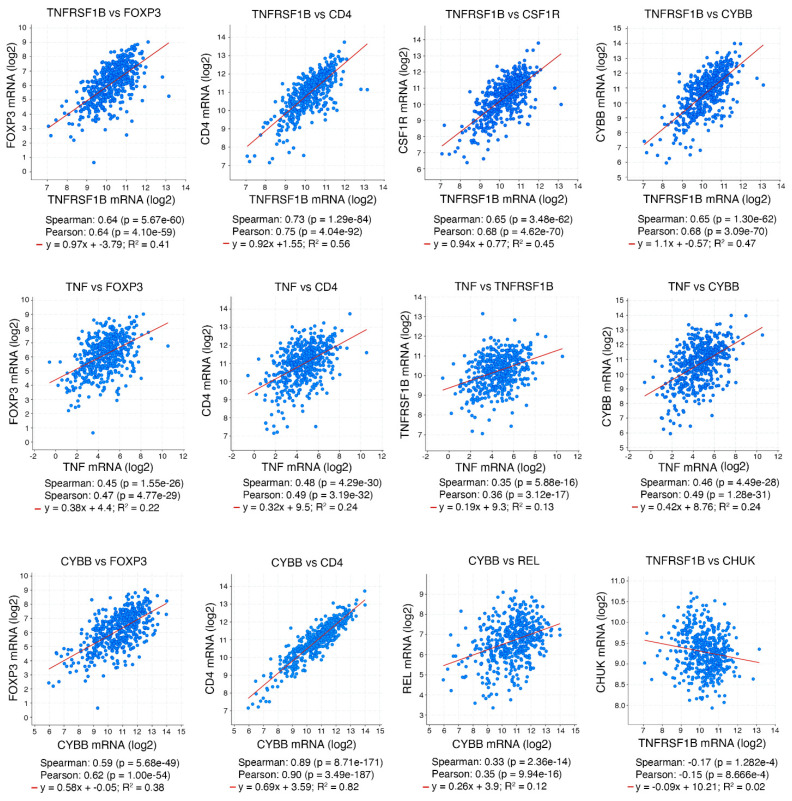
Expression correlations of genes related to FOXP3 Treg cell induction in human lung ADCs (cBioPortal for Cancer Genomics, TCGA, PanCancer Atlas).

TME regulates tumorigenesis. We detected a correlated increase in numbers of macrophages and Foxp3^+^ Treg cells in *Ikka^∆LU^;Kras^G12D^* ADCs that were associated with increased tumor burdens and reduced CD8 cell numbers compared to *Kras^G12D^* ADCs [11]. Macrophage-produced reactive oxygen species (ROS) are required for Treg cell induction [11]. Depleting macrophages decreased Treg cell numbers and inhibited lung ADC development, and depleting Treg cells indeed dampened lung ADC development in *Ikka^∆LU^;Kras^G12D^* mice, indicating that increased Treg cell number is a mechanism for ADC progression. Furthermore, macrophage-produced ROS and TNF stimulated TNFR2 and NF-κB activation in CD4 T cells, which converted CD4 T cells to Treg cells. Inhibition of NF-κB c-Rel decreased Treg cell induction in vivo and reduced lung tumorigenesis. In addition, *Ikka^∆LU^;Tnfrsf1b^-/-^;Kras^G12D^* mice reduced lung ADC burdens with reduced Treg cell numbers compared to *Ikka^∆LU^;Kras^G12D^* mice. Intratracheal injection of a lung ADC cell line to *Tnf^-/-^* mice showed reduced lung ADC burdens, which were associated with decreased Treg cell induction, compared to WT mice [11], confirming that TNF/TNFR2/NF-κB is required for Treg cell induction in TME. Interestingly, the co-culture of WT macrophage and *Tnfrsf1b^-/-^* CD4 T cells treated with TNF showed a partial reduction in Treg cell numbers compared to WT CD4 T cells in the co-culture, suggesting that TNFR1 may contribute to Treg induction in the absence of TNFR2 [11]. Because macrophage-ROS and TNF/TNFR2/NF-κB play a role that affects Treg cell induction associated with ADC burdens in *Ikka^∆LU^;Kras^G12D^* mice, we further analyzed the expression of the associated genes to determine the relevance of this pathway in human lung ADC cohorts (cBioPortal for Cancer Genomics). Patients with lung ADCs expressing reduced *CHUK* and increased *TNFRSF1B* died earlier than lung ADC patients expressing increased *CHUK* and decreased *TNFRSF1B* [11]. Furthermore, human lung ADC cohorts showed that *TNFRSF1B* expression levels were highly correlated with *FOXP3*, *CD4*, *CSF1R*, and *CYBB* expression; that *TNF* expression levels were highly correlated with *FOXP3*, *CD4*, *TNFSF1B*, and *CYBB* expression; and that *CYBB* expression levels were highly correlated with *FOXP3*, *CD4*, and *REL* expression (Figure 3). On the other hand, *TNFRSF1B* expression levels were opposite to *CHUK* expression levels (cBioPortal for Cancer Genomics). Reintroduction of IKKα repressed lung tumorigenesis in this animal system and IKKα reduction upregulated the expression of TNF, CSF1, CCL22, and IL-23A. CSF1 and CCL22 recruited macrophages and IL-23A blocked CD8 T-cell infiltration [49]. Thus, the increased cytokines generated a milieu to facilitate the development of an iTME. The tight correlation between the ROS/TNF/TNFR2/NF-κB pathway and Treg cell induction in human and mouse lung ADCs indicates the medical significance of this finding.

## 4. Conclusions and Therapeutic Perspective

**Figure 4:**
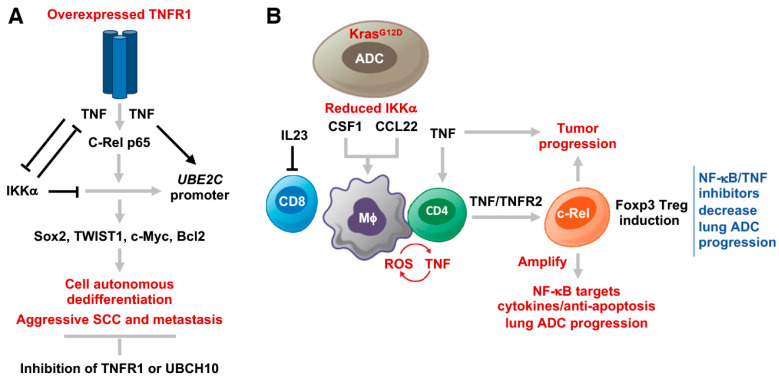
(**A**) and (**B**) The mechanisms of TNFR1 and TNFR2 in the promotion of lung SCC metastasis and lung ADC progression.

Overall, TNFR1 and TNFR2 have been demonstrated to promote aggressive lung SCC development and metastasis and accelerate lung ADC progression through distinct mechanisms in different mouse models (Figure 4A,B). In addition, *Ikka* ablation is a cancer driver in these lung cancer animal models, and reintroduction of IKKα inhibited the ADC and SCC development. Although IKKα does not directly regulate TNFR1 or TNFR2 expression, we found that IKKα inhibited the expression of the targets of TNFR1 and TNFR2 in carcinogenesis. However, a puzzling relationship between IKKα and IKK activity as downstream targets of TNFRs in carcinogenesis remains to be investigated in the future.

Several literature reviews have discussed the multiple mechanisms by which TNF and TNFRs regulate immune responses and lung cancer development [50,51]. Of note, TNF has been suggested to inhibit tumor development, but phase I and phase II clinical trials for treating cancer patients with TNF were not successful [52,53,54]. Here, our studies provided new insights into the roles of TNF, TNFR1, and TNFR2 in promoting lung cancer development, including the following: (1) *Tnfr1* deletion prevented lung SCC development in *KA/KA;Tnfr1^-/-^* mice compared to *KA/KA* mice; (2) silencing TNFR1 in KAL^LU+^ lung SCC cells reduced stemness and Bcl2 anti-apoptotic protein levels but increased differentiation markers and E-cadherin that is expressed on the surface of well-differentiated SCC compared to parental KAL^LU+^ cells, and TNF regulated the differentiation and EMT markers in SCC cells [10,55]; (3) KAL^LU+^ cells expressing downregulated TNFR1 inhibited lung SCC burdens and blocked the SCC transition from well-differentiated lung SCCs to spindle cell carcinomas, a final stage of SCC, and metastasis compared to the parental KAL^LU+^ cell-induced lung tumors in WT and *KA/KA* mice; (4) silencing TNF in KAL^LU+^ cells inhibited lung tumor development compared to parental KAL^LU+^ cells in WT and *KA/KA* mice; (5) the numbers of tumor infiltrating Treg cells and lung ADC burdens were reduced in *Tnfr2^-/-^;Kras^G12D^;Ikka^∆LU^* mice compared to *Kras^G12D^;Ikka^∆LU^* mice; and (6) the lung ADC burden induced by injected lung ADC cells and tumor infiltrating Treg numbers were significantly lower in *Tnf^-/-^* mice than in WT mice [10,11]. These findings suggest that TNF, TNFR1, and TNFR2 regulate intratumoral oncogenic pathways and modulate TME. Thus, these molecules may be considered to be potential therapeutic targets. To date, the commercial antibodies, antagonists, and small-molecule inhibitors against TNF, TNFR, TNFR1, and TNFR2 have been successfully used to treat several human autoimmune diseases, such as rheumatoid arthritis, inflammatory bowel disease, and psoriasis [56,57,58,59,60]. Taken together, to increase the efficacy of anti-tumor treatment with anti-TNFR-related reagents, it may be important to examine the alterations in TNFR1, TNFR2, and TNF levels and Treg cell numbers in human lung tumors. Then, the anti-TNFR1/TNF or anti-TNFR2/TNF therapeutic approach could be used to treat those selected patients with lung cancers expressing the relevant markers.

## Data Availability

Not applicable.
